# Frequency-Domain Features and Low-Frequency Synchronization of Photoplethysmographic Waveform Variability and Heart Rate Variability with Increasing Severity of Cardiovascular Diseases

**DOI:** 10.3390/biomedicines12092088

**Published:** 2024-09-12

**Authors:** Anton R. Kiselev, Olga M. Posnenkova, Anatoly S. Karavaev, Vladimir A. Shvartz, Mikhail Yu. Novikov, Vladimir I. Gridnev

**Affiliations:** 1 Coordinating Center for Fundamental Research, National Medical Research Center for Therapy and Preventive Medicine, 10 Petroverigsky Pereulok, Bld. 3, Moscow 101990, Russia; 2 Institute of Cardiology Research, Saratov State Medical University, Saratov 410012, Russia; 3 Department of Dynamic Modeling and Biomedical Engineering, Saratov State University, Saratov 410012, Russia; 4 Department of Surgical Treatment for Interactive Pathology, Bakulev National Medical Research Center for Cardiovascular Surgery, Moscow 121552, Russia

**Keywords:** heart rate variability, photoplethysmographic waveform variability, frequency-domain indices, low-frequency synchronization, signal analysis

## Abstract

*Objective*—Heart rate variability (HRV) and photoplethysmographic waveform variability (PPGV) are available approaches for assessing the state of cardiovascular autonomic regulation. The goal of our study was to compare the frequency-domain features and low-frequency (LF) synchronization of the PPGV and HRV with increasing severity of cardiovascular diseases. *Methods*—Our study included 998 electrocardiogram (ECG) and finger photoplethysmogram (PPG) recordings from subjects, classified into five categories: 53 recordings from healthy subjects, aged 28.1 ± 6.2 years, 536 recordings from patients with hypertension (HTN), 49.0 ± 8.8 years old, 185 recordings from individuals with stable coronary artery disease (CAD) (63.9 ± 9.3 years old), 104 recordings from patients with myocardial infarction (MI) that occurred three months prior to the recordings (PMI) (65.1 ± 11.0 years old), and 120 recordings from study subjects with acute myocardial infarction (AMI) (64.7 ± 11.5 years old). Spectral analyses of the HRV and PPGV were carried out, along with an assessment of the synchronization strength between LF oscillations of the HRV and of PPGV (synchronization index). *Results*—Changes in all frequency-domain indices and the synchronization index were observed along the following gradient: healthy subjects → patients with HTN → patients with CAD → patients with PMI → patients with AMI. Similar frequency-domain indices of the PPGV and HRV show little relationship with each other. *Conclusions*—The frequency-domain indices of the PPGV are highly sensitive to the development of any cardiovascular disease and, therefore, are superior to the HRV indices in this regard. The S index is an independent parameter from the frequency-domain indices.

## 1. Introduction

The heart rate variability (HRV) is the most commonly used phenomenon to study the autonomic regulation of the cardiovascular system (mainly, cardiac chronotropic control) [1]. The frequency-domain indices of the HRV have been well studied in healthy people and various patients [1]. Short-term HRV recordings are characterized by three main spectral bands: very-low-frequency (VLF; 0.003–0.04 Hz), low-frequency (LF; 0.04–0.15 Hz), and high-frequency (HF; 0.15–0.4 Hz) components [1,2]. Oscillations in the high-frequency band of the spectrum are caused by the cardiac activity of the vagus nerve and the effect of breathing on the heart rate (HR) [1]. LF oscillations reflect baroreflex function [3,4,5] and, to a lesser extent, both sympathetic and cardiac vagal activity [4,5,6,7]. Lower values of the power in the LF spectrum of the HRV were observed in patients with reduced baroreflex sensitivity (BRS), regardless of cardiac innervation [2]. The power of the VLF spectral band of the HRV is a measure of various humoral and local factors, as well as sympathetic activity, affecting the heart rate (HR) [2]. Currently, the features of LF and HF oscillations in the HRV are increasingly attracting the attention of researchers. Sympathovagal balance is often described by the LF/HF band ratio in the HRV spectrum [1]. Yet, it is probably more accurate to interpret this LF/HF band ratio as a relationship between baroreflex function and respiratory vagal modulation of the HR. In healthy subjects at rest, the LF/HF band ratio of the HRV ranges from 1 to 2 [2]. A decrease in the HRV indices is consistently observed in patients who have suffered myocardial infarction (MI), hypertension (HTN), diabetic neuropathy, heart failure, etc. [1]. 

Blood pressure variability (BPV) is the second most important phenomenon in assessing the autonomic regulation of the cardiovascular system. BPV is primarily caused by peripheral vasomotor tone, which is not directly related to heart rate control. Fluctuations in peripheral blood flow can be studied based on photoplethysmographic waveform variability (PPGV), which characterizes beat-to-beat changes in PPG amplitude. The photoplethysmogram (PPG) is most often recorded on the fingers [8]. The PPG signal of the finger contains data on both the microvasculature and blood filling of the digital arteries [9,10]. Kamshilin et al. argued about the close connection between reflection-mode PPG and blood pressure (BP) signals, based on their own physiological model [10]. The spectrum of PPGV reveals oscillations with a frequency in bands similar to those specified for HRV [11]. There is an opinion that HF oscillations in the PPGV primarily reflect the mechanical effect of breathing, while LF oscillations are associated with the sympathetic regulation of vascular tone [12,13,14]. Gonzalez et al. suggested that the oscillations of the PPGV and BPV are similar, albeit not identical [15], yet it is possible that they characterize similar mechanisms of vascular regulation [16]. It has been shown that the LF oscillations of HRV and PPGV can be synchronized with each other [17], which may be due to the peculiarities of the interaction of slow mechanisms in autonomic regulation of cardiovascular function. This synchronization is reduced in patients with cardiovascular diseases and can be modified by medical treatment [17]. 

To date, the features of spectral assessments of the PPGV of the finger have been poorly studied. We previously revealed that the effect of breathing on the HF band of the PPGV spectrum in healthy subjects is lower compared to the HRV spectrum [11]. A significant difference between the PPGV and HRV spectra was also shown in the LF/HF ratio: the power of the LF band predominated over the HF spectral band (the LF/HF ratio in PPGV usually ranged from 4 to 11) [11]. 

The goal of our study was to compare the frequency-domain features and LF synchronization of the PPGV and HRV with increasing severity of cardiovascular conditions.

## 2. Materials and Methods

### 2.1. Ethical Approval

The study was approved by the Ethics Committee of Saratov State Medical University, Saratov, Russia, and written informed consent was obtained from all participants. All procedures involving human participants were performed in accordance with the ethical standards of the institutional research committee and the 1964 Declaration of Helsinki and its later amendments.

### 2.2. Dataset

Our study included 998 electrocardiogram (ECG) and finger PPG recordings from subjects classified into five categories: 53 recordings from healthy subjects, aged 28.1 ± 6.2 years, 536 recordings from patients with HTN, aged 49.0 ± 8.8, 185 recordings from individuals with stable coronary artery disease (CAD) (63.9 ± 9.3 years old), 104 recordings from patients with MI occurring three months prior to the records (PMI) (65.1 ± 11.0 years old), and 120 recordings from study subjects with acute myocardial infarction (AMI) (64.7 ± 11.5 years old). Spectral analyses of HRV and PPGV were carried out, along with an assessment of the synchronization strength between LF oscillations of HRV and of PPGV (synchronization index). 

We used databases of biological signals and clinical metadata from our previous studies of autonomic control of the cardiovascular system in different types of study subjects: healthy subjects [11,18], patients with HTN [19], individuals with stable CAD [20,21], patients with PMI [22], and subjects with AMI [22]. An additional study was based on the clinical practice at the Institute of Cardiology Research, Saratov State Medical University. Synchronous recordings of three biological signals were selected for further analysis: ECG, finger PPG, and the respiratory signal.

We used the following criteria to include the above-listed recordings in our study:

(i) Confirmed clinical status or diagnosis (healthy clinical status, HTN, CAD, PMI, or AMI) in accordance with current clinical guidelines;
(ii) Age from 18 to 80 years;
(iii) Obtained written informed consent to use the data for further analysis.

Recordings were not included in our study if patients met the following criteria:

(i) HR disorders that interfere with HRV analysis;
(ii) Valvular heart disease;
(iii) Endocrine pathology, with the exception of compensated diabetes (only for groups of patients with CAD, PMI, or AMI);
(iv) Endocrine pathology, including diabetes mellitus (only for groups of healthy individuals and patients with HTN);
(v) Secondary HTN;
(vi) Disorders of peripheral microcirculation;
(vii) Chronic gastrointestinal diseases (hepatitis, duodenitis and cholecystitis), chronic kidney diseases and other chronic diseases in the acute stage;
(viii) Subclinical organ damage in accordance with the guidelines [23] (solely for groups of healthy subjects and patients with HTN);
(ix) MI less than a year ago (only for groups of healthy individuals, patients with CAD, and patients with HTN);
(x) Other cardiovascular diseases (only for groups of healthy individuals and patients with HTN).

Inclusion and exclusion criteria were determined based on clinical metadata. We did not take into account the characteristics of pharmacological treatment in patient groups.

Our study ultimately included 998 recordings of three types of biological signals from study participants divided into five categories:

(i) 53 recordings from healthy subjects (38 male; 71.7%), aged 28.1 ± 6.2 years (data presented as mean and standard deviation, M ± SD);
(ii) 536 recordings from patients with HTN (176 male; 32.8%), aged 49.0 ± 8.8 years;
(iii) 185 recordings from patients with CAD (110 male; 59.5%), aged 63.9 ± 9.3 years,
(iv) 104 recordings from patients with PMI (60 male; 57.7%), aged 65.1 ± 11.0 years,
(v) 120 recordings from patients with AMI (70 male; 58.3%), aged 64.7 ± 11.5 years.

### 2.3. Signal Characteristics

For all subjects who were examined in the afternoon hours on an empty stomach, in conditions of spontaneous breathing, we simultaneously recorded ECG, PPG from the middle finger of the right hand, and the respiratory signal. The signals were recorded in a quiet room with controlled temperature. The signal sampling frequency was 250 Hz, the resolution was 14 bits. The respiratory signal was recorded to monitor the evenness of breath. All experimental signals were recorded using an electroencephalograph analyzer EEGA-21/26-Encephalan-131-03 (Medicom MTD LLC, Taganrog, Russia). Series of recorded signals during forced breathing and breath holding were excluded from the analysis. Only ECG and PPG recordings without artifacts, extrasystoles, and pronounced trends were left for further analysis. For patients with PMI or AMI, we conducted 10 min recordings of biological signals exclusively at rest (supine position of the subject’s body). 

In healthy subjects, patients with HTN and patients with CAD, we recorded biological signals for 20 min during the passive head-up tilt test. The latter stimulates orthostatic adaptation responses in cardiovascular autonomic regulation [24,25,26]. In these groups, ECG, PPG, and respiration were recorded at each stage of the passive head-up tilt test. Orthostatic stress (during the passive head-up tilt test) is often employed as an experimental condition challenging the baroreflex and other mechanisms of cardiovascular autonomic control. For example, the passive head-up tilt test is used to study sympathetic activity [27] and the factors influencing autonomic control [28], as well as for the diagnosis of syncope [29]. The algorithm of the passive head-up tilt test used in our study included the following steps:

(i) During the preliminary stage, lasting 10 min, the study subject lay in a horizontal position without signal recording;
(ii) Signals were recorded for 10 min with the subject’s body in a supine position;
(iii) The subject was passively placed in an upright position with a tilt angle of about 80°. To exclude transient processes, signals were not recorded for 5 min;
(iv) Signals were recorded for 10 min with the subject’s body in an upright position.

### 2.4. Data Preprocessing

To analyze HRV, a sequence of normal-to-normal interbeat intervals (NN) measured in milliseconds (the time elapsed between two consecutive R peaks on an ECG with normal sinus rhythm) was extracted from the ECG. Due to HRV, the NN interval measurements are not equidistant in time. To transform the non-equidistant signal into an equidistant one, we used cubic spline interpolation, low-pass filtering with a 0.5 Hz cutoff frequency, and then resampling at regular intervals with a sampling frequency of 4 Hz (according to R.M. Baevsky et al. [30]). The resulting equidistant sequence of NN intervals was used for further processing.

No preprocessing of the PPG and respiratory signals was performed.

### 2.5. Spectral Analysis of Heart Rate Variability

Spectral analysis of HRV was performed in compliance with the methodological guidelines adopted in Russia [30,31,32], which are consistent with the European/American guidelines [1]. Data segments of 300 s duration were used for spectral analysis. Linear trends were removed, and the power spectral density was estimated using the Welch algorithm based on the fast Fourier transform using segments of 256 data points with 50% overlap and a Hanning window. 

We calculated the following frequency-domain HRV indices: LF (power spectral density integrated over the 0.04–0.15 Hz band and measured in ms^2^), HF (power spectral density integrated over the 0.15–0.4 Hz band and measured in ms^2^), LF/HF ratio, TP (short for total power; power spectral density integrated over the 0–0.4 Hz band and measured in ms^2^), LF% (LF/TP ratio measured in percentage), and HF% (HF/TP ratio measured in percentage). In this study, we denoted the spectral HRV estimates with the subscript ‘_HRV_’ (e.g., HF_HRV_). 

The VLF band was not included in our analysis to avoid confounding results, since we used short-term ECG recordings [1]. 

In addition to frequency-domain indices, we analyzed the means of the heart rate (HR) measurements.

### 2.6. Spectral Analysis of Photoplethysmographic Waveform Variability

PPGV power spectra were calculated directly from PPG signals using the Welch’s method [33] in two-minute windows, with a one-minute shift. A critical power value was calculated, above which the spectral components were considered statistically significant (*p* = 0.05). For this purpose, we tested the statistical hypothesis of normal bandwidth-limited noise (0.04–2 Hz) using surrogate data. Before the study, we compared the estimates of the power spectral density for different window lengths. As a result, we chose the above parameters because they comply with the official guidelines adopted in Russia for the spectral assessment of HRV signals [30] and give satisfactory results in estimating the power in the frequency band when comparing groups based on PPG signals, although promising data analysis methods based on parametric spectral assessment or nonlinear filtering (e.g., via empirical mode decomposition) are known [34].

Then, using an approach similar to that used for HRV analysis [1,30], we calculated the following spectral indices of PPGV for these spectra: LF%, HF%, and LF/HF. In this study, we denoted the spectral PPGV estimates with the subscript ‘_PPGV_’ (e.g., HF_PPGV_). 

One of the problems with using PPG is the difficulty of interpreting the absolute values of the PPG waveform. The signal at the output of the optical PPG sensor is proportional to an unknown coefficient, the value of which depends on a number of factors, such as the optical features of the subject’s skin, BP, sensor placement, electrical and optical characteristics of the sensor, as well as room illumination and temperature. Absolute PPGV values are measured in conventional units (CUs), which are the values of discrete samples of the PPG waveform, proportional to the signal at the output of the optical sensor. Since the proportionality coefficient between volumetric blood flow and CU is unknown, the interpretation of the absolute values of the LF, HF, and TP indices is problematic. In this paper, we do not use these spectral indices. However, the dimensionless ratios (LF/HF, LF%, and HF%) have similar meaning as for HRV. Previously, we successfully used the described approach to PPGV analysis in studies involving adult patients [35].

### 2.7. Assessment of Synchronization between the Low-Frequency Oscillations in Heart Rate Variability and Photoplethysmographic Waveform Variability 

To estimate the synchronization of low-frequency oscillations in HRV and PPGV, we applied the method proposed by us recently [17]. The S index expresses the relative time (in percent) of synchronization between the considered LF oscillations. The calculation of the S index consists of several steps: the signals of the PPG and RR interval signals are filtered using a 0.05–0.15 Hz bandpass filter; the oscillation phases are calculated from the filtered signals; the difference between the resulting phases is calculated; an automated algorithm for detecting horizontal sections of signal phase differences is applied; the horizontal sections correspond to the frequency synchronization intervals between the signals; and the total duration of the horizontal sections is calculated and divided by the total duration of the signals to estimate the S index.

### 2.8. Statistical Analyses

Continuous variables are presented as medians with lower and upper quartiles: Me (LQ, UQ). For some continuous variables, the minimum (Min) and maximum (Max) values are presented.

We used the Shapiro–Wilk test to check whether the data were approximately normally distributed. Since most of the data were non-normal, further analysis was performed using nonparametric statistical methods. For pairwise comparisons of variables (at rest and after the passive head-up tilt test) within a group of subjects, we used the Wilcoxon test. For pairwise comparisons of a variable between groups of subjects, we used the Mann–Whitney test. We also used the Kruskal–Wallis analysis of variance with post hoc comparisons of mean ranks for multiple comparisons of a variable between groups of subjects. Pairwise associations between continuous variables were assessed using Spearman’s correlation coefficient (R).

The obtained estimates were considered statistically significant at *p* < 0.05.

## 3. Results

### 3.1. Comparison of Cardiovascular Autonomic Indices between Study Groups 

We revealed multiple statistically significant differences (Kruskal–Wallis test: *p* < 0.001) between the subject groups for all studied autonomic system parameters, except for resting HR (Table 1). In Table 1, we present the results of the post-hoc analysis of differences and similarities between groups using a color legend. With increasing severity of the clinical status of subjects (healthy subjects → patients with HTN → patients with CAD → patients with PMI → patients with AMI), the values of some indices (S index, HF_HRV_, LF_HRV_, TP_HRV_, LF%_HRV_, LF/HF_HRV_ in upright position, LF%_PPGV_, and LF/HF_PPGV_) decreased, while HF%_PPGV_ increased. Note that HF%_HRV_ and the resting LF/HF_HRV_ were similar in healthy subjects and both groups of patients with MI; these indices exhibited the lowest values in patients with HTN and CAD (only for HF%_HRV_ at rest) (Table 1). Any cardiovascular pathology (HTN, CAD, MI) was associated with a sharp change (vs. healthy people) in the following autonomic indices: the S index, LF%_HRV_, LF/HF_HRV_ in the upright position, and all spectral parameters of PPGV (Table 1). More pronounced differences were found for the spectral parameters of PPGV. In particular, we found that, compared with healthy people, the respiratory effects on PPGV (assessed by HF%_PPGV_) increased threefold to fourfold in patients with HTN and up to eightfold in patients with CAD or MI. Against this background, in all patients, the contribution of the sympathetic outflow to the PPGV (estimated by LF%_PPGV_) decreased sevenfold to twentyfold, compared with healthy individuals.

### 3.2. Orthostatic Dynamics of Cardiovascular Autonomic Indices

We established significant (*p* < 0.05) orthostatic changes in most of the studied autonomic nervous system indices in all study groups (these differences are marked with an asterisk in Table 1).

During the passive head-up tilt test, we observed a decrease in the following indices: HF_HRV_, HF%HRV, LF_HRV_ (solely in patients with CAD), TP_HRV_ (except for healthy individuals), and LF/HF_PPGV_ (in both healthy individuals and patients with HTN) (Table 1).

The passive head-up tilt test yielded an increase in the values of the following indices: the HR, LF%_HRV_ (only in healthy individuals and patients with hypertension), LF/HFHRV, and HF%PPGV (only in healthy individuals and patients with HTN) (Table 1).

LF%_PPGV_ did not change in all subjects. The S index exhibited opposite changes in healthy subjects and patients: it increased after the passive head-up tilt test in healthy subjects and decreased in patients with cardiovascular diseases (Table 1).

### 3.3. Comparison of Spectral Indices of Heart Rate Variability and Photoplethysmography Waveform Variability

**Figure 1:**
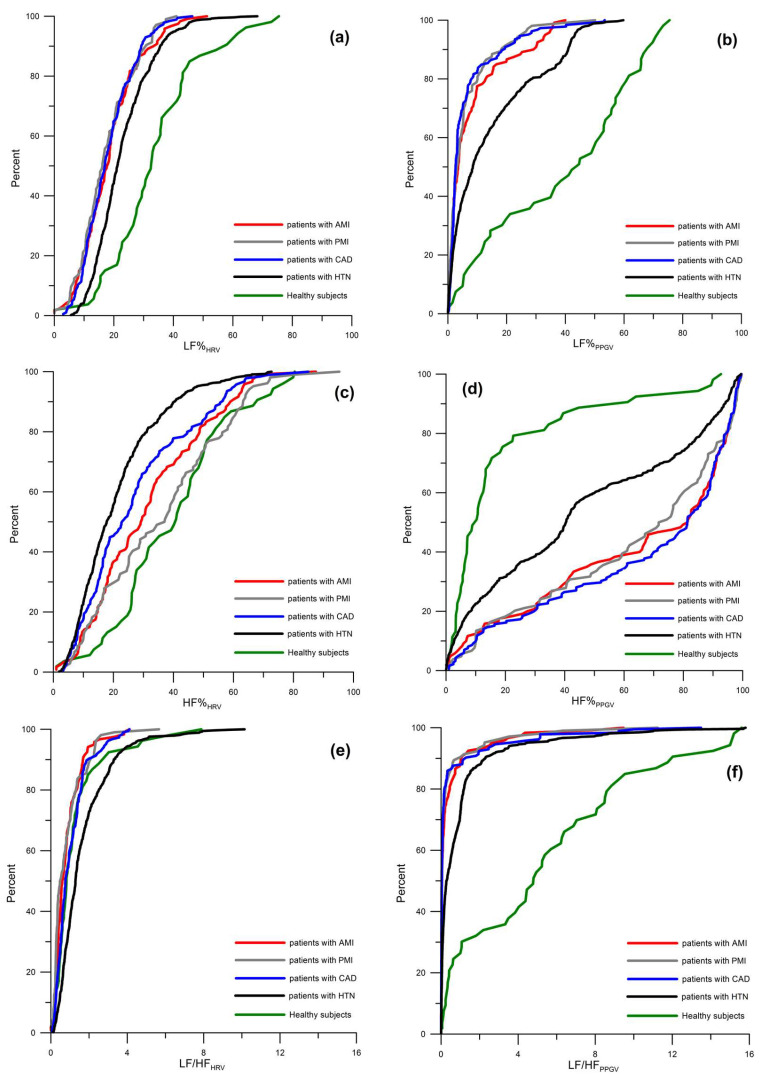
Cumulative distribution curves of frequency-domain indices (LF%, HF%, and LF/HF) of heart rate variability (HRV) (**a**,**c**,**e**) and photoplethysmographic waveform variability (PPGV) (**b**,**d**,**f**) in healthy subjects, patients with hypertension (HTN), patients with coronary artery disease (CAD), patients with previous myocardial infarction (PMI), and patients with acute myocardial infarction (AMI).

In healthy subjects, we detected no differences between LF%_HRV_ and LF%_PPGV_ in the supine position (*p* = 0.183) (Table 1; Figure 1a,b), while LF%_HRV_ was slightly greater than LF%_PPGV_ after the passive head-up tilt test (*p* = 0.017) (Table 1). HF%_HRV_ was significantly higher than HF%_PPGV_ at rest (*p* < 0.001) (Table 1; Figure 1c,d) and lower after the passive head-up tilt test (*p* = 0.018) (Table 1). In the supine position, LF/HF_HRV_ was significantly lower than LF/HF_PPGV_ (*p* < 0.001) (Table 1; Figure 1e,f), while in the upright body position, both had similar values (*p* = 0.251) (Table 1). 

In patients with cardiovascular diseases, we observed statistically significant differences (*p* < 0.001) between the studied spectral indices of HRV and PPGV (LF%, HF% and LF/HF) in both body positions. The distributions of these parameters in the study groups at rest are shown in Figure 1. Thus, the spectrum of the PPGV in patients is characterized by a significantly lower LF/HF and LF%, and a higher HF%, compared with the HRV spectrum (Figure 1; Table 1).

### 3.4. Associations between Cardiovascular Autonomic Indices

For all subjects in our study, we investigated the correlations between the studied cardiovascular autonomic indices (Table 2). We identified a number of trivial correlations (TP with LF and HF; LF/HF with LF% and HF%). The S index had minimal and non-significant correlation only with some of the indices. The HR had a moderate correlation only with the spectral indices of HRV, measured in ms^2^.

We discovered a minimal and non-significant correlation between the following pairs of HRV and PPGV indices: HF%_PPGV_ and HF%_HRV_, HF%_PPGV_ and LF%_HRV_, HF%_PPGV_ and LF/HF_HRV_, LF%_PPGV_ and LF%_HRV_, LF%_PPGV_ and LF/HF_HRV_, LF/HF_PPGV_ and HF%_HRV_, LF/HF_PPGV_ and LF%_HRV_, as well as LF/HF_PPGV_ and LF/HF_HRV_ (Table 2).

We examined multiple associations between the S index and other autonomic indices based on backward stepwise regression analysis. The best regression model contains two independent variables (LF%_HRV_ and LF/HF_PPGV_) to predict the S index (multiple R = 0.34, R^2^ = 0.12, *p* < 0.001). The beta coefficients for the independent variables in the constructed model were as follows: LF%_HRV_ = 0.157 and LF/HF_PPGV_ = 0.210.

## 4. Discussion

### 4.1. Photoplethysmographic Waveform Variability for Assessing Cardiovascular Autonomic Control

With increasing severity of arterial disease, the peripheral pulse usually becomes attenuated, slower, and weaker [36,37,38]. This fact determines the clinical applicability of PPG for the detection of lower limb diseases and other vascular dysfunctions. That is why, most of the published works seem to have focused on pulse rate variability (PRV) assessed by PPG. PRV is also commonly used as an acceptable surrogate for HRV [39,40,41,42,43,44]. However, such an interpretation of PRV has known limitations [45,46]. 

PPGV provides qualitatively different information, characterizing oscillations of peripheral blood flow. Infrared PPGV shows two main spontaneous oscillations: LF oscillations at a frequency of 0.1 Hz and respiration-related components at a frequency of 0.25 Hz [12]. Nitzan et al. proposed PPGV as a potential method for assessing the autonomic nervous system [47], along with the local control of tissue microcirculation. It is known that the sympathetic baroreflex function, based on the activity of muscle sympathetic nerves, controls systemic vascular resistance on a beat-to-beat basis [48]. The relationship between LF oscillations in PPGV and sympathetic activity was previously confirmed by many authors [12,49,50,51,52,53]. Colombo et al. showed that PPGV values were associated with changes in sympathetic outflow directed to vessels and sympathovagal balance modulating heart rate [54]. A strong baroreflex effect on peripheral blood volume was also noted by Martinez-Garcia et al. [55]. The neural nature of LF oscillations in PPGV is supported by the fact that diabetic neuropathy causes a bilateral difference in these oscillations [47,56]. 

Grinevich et al. suggested that oscillations with a frequency of about 0.1 Hz in arterial blood flow may arise under the influence of hydrodynamic factors, without the participation of autonomic control [57]. This hypothesis, which is inconsistent with our studies, presents a considerable interest and requires careful study. Moreover, Kamshilin et al. proposed the original early physiological model capturing the close relationship between PPG and BP signals [10]. Therefore, we believe that PPGV indices may be related to pulse wave variability to some extent.

Bernardi et al. showed a high coherence between the LF oscillations obtained in BPV, in PPGV, and in HRV, which was not related to respiration or pulse rate, suggesting a common neural, nonlocal origin [12]. Sympathetic vasoconstrictor outflow is regulated by the arterial baroreflex in a beat-to-beat mode [58]. Yet, the phase between PPGV and BPV is positive around 0.1 Hz, indicating that LF oscillations are not passively transmitted into the PPG signal from large arteries [12]. Passive transmission of respiratory blood oscillations into the PPG signal assessed in the HF spectral band of PPGV was also reported [12,13,14]. The presented facts are of interest, given the influence of both the microvasculature and the blood filling of the digital arteries on the finger PPG signal, which was reported by Rhee et al. [9]. Thus, LF oscillations in PPGV can be used as a measure of baroreflex control of the BP via the sympathetic modulation of vascular resistance. 

**Figure 2:**
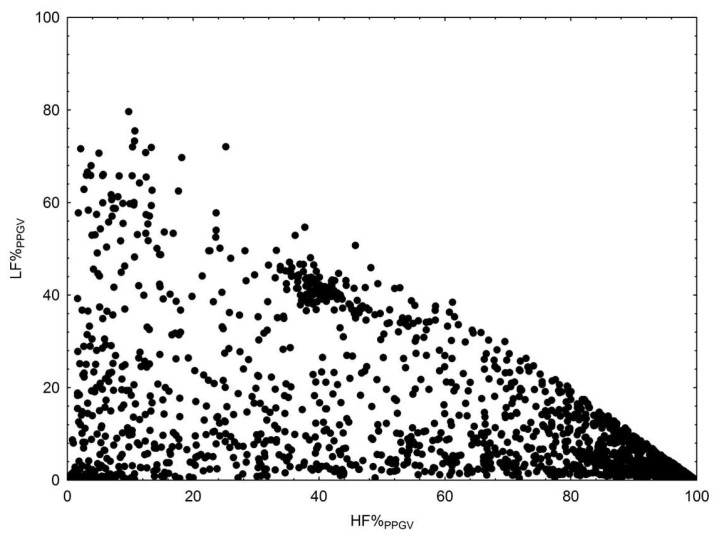
Scatterplot of LF%_PPGV_ vs. HF%_PPGV_.

In our study, all spectral indices of PPGV (LF%, HF%, and LF/HF) were highly sensitive to the development of any cardiovascular disease, surpassing the HRV indices in this regard (Table 1). In all patients with cardiovascular diseases, vascular sympathetic activity was significantly reduced in PPGV, and the proportion of passive oscillations associated with breathing was increased. Associations between HF%_PPGV_ and LF%_PPGV_ are shown in Figure 2 (R = −0.43, *p* < 0.001). It is possible that changes in the properties of the arterial bed in patients with cardiovascular diseases lead to a decrease in the vascular bed reactivity in response to the sympathetic outflow (through the vasoconstrictor nerves) and a reciprocal increase in respiratory effects on the peripheral blood flow. The relationship between the effects of sympathetic outflow and respiratory influences on the finger PPG signal, which progressively decreases with increasing severity of cardiovascular disease (healthy → HTN → stable CAD → PMI → AMI), can be estimated through the LF/HF ratio. These facts highlight the potential clinical significance of PPGV for the early detection of cardiovascular autonomic disorders associated with any cardiovascular disease. Other authors also confirmed the promise of using PPGV spectral analysis for testing the autonomic nervous system and endothelial function [59].

It is worth noting that similar spectral indices of PPGV and HRV (HF%_PPGV_ and HF%_HRV_, LF%_PPGV_ and LF%_HRV_, as well as LF/HF_PPGV_ and LF/HF_HRV_) are weakly related (Table 2). Thus, we conclude that these two pathways of cardiovascular autonomic control (conductance of the heart and of total vascular conductance) are independent of each other. However, it is necessary to take into account the modeling study by Ursino and Magosso, who reported a relationship between a decrease in the intensity of LF noise affecting vascular conductance and a reduction in the magnitude of the LF component of the HRV spectrum [60]. We have not yet found any significant experimental evidence supporting this hypothesis. 

The superiority of PPGV parameters over HRV in terms of detecting any cardiovascular disease may indicate that the dysfunction of blood flow regulation is more involved in the pathogenesis of these diseases than heart rate regulation. In particular, this may be due to differences in the influence of multiple factors (sympathetic overdrive, external stressors, etc.) involved in the development of cardiovascular diseases [61] on the mechanisms regulating heart rate and blood flow.

### 4.2. Synchronization between Low-Frequency Oscillations in Heart Rate Variability and Photoplethysmographic Waveform Variability

The cardiac baroreflex is the main mechanism of the closed interaction between the BP and the HR, which mutually influence each other [62,63,64]. This mechanism maintains the BP near a conventional value. The main role in the baroreflex control of the BP belongs to the carotid and aortic baroreceptors, which cause changes in the activity of the neural centers of cardiovascular regulation [65]. In order to control the BP, these neural adjustments affect both the heart and the blood vessels accordingly [65,66]. 

Changes in the biophysical properties of the BP/HR interaction (time delay, causality) are associated with impaired cardiovascular regulation (e.g., linked with orthostatic syncope) [67]. The function of the cardiac baroreflex is mirrored in the main part of the LF oscillations in HRV [3,4,5]. LF oscillations in PPGV can also be directly related to the baroreflex regulation of the BP through the total vascular conductance, given that the PPG signal contains significant information about the blood filling of the digital arteries [9]. 

**Figure 3:**
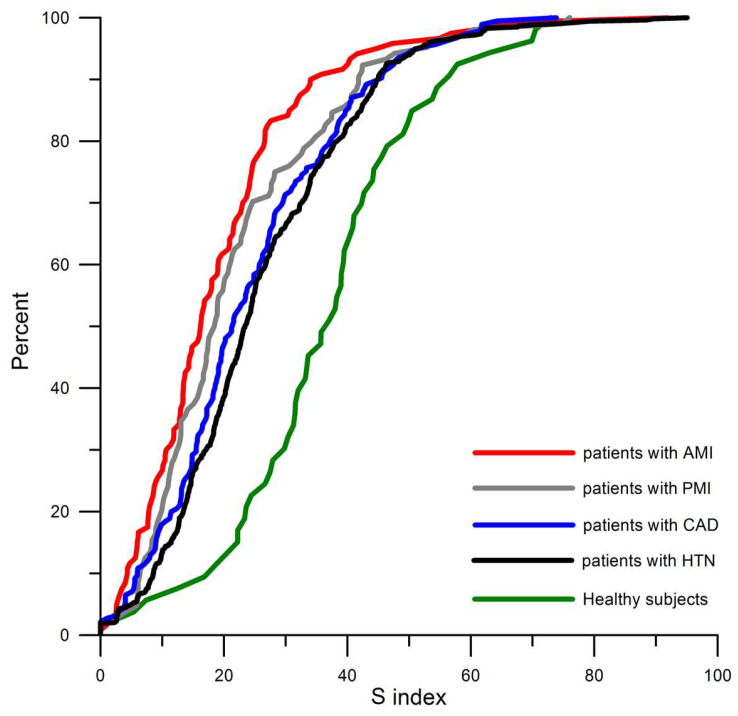
Cumulative distribution curves of the S index in healthy subjects, patients with hypertension (HTN), patients with coronary artery disease (CAD), patients with previous myocardial infarction (PMI), and patients with acute myocardial infarction (AMI).

We have previously shown that LF oscillations in PPGV and HRV can be synchronized with each other [17]. This synchronization may be the result of the interaction of autonomic mechanisms regulating the cardiovascular system (heart and vascular bed) in order to control the BP. In the present study, we confirmed our previous results on the decrease in the quality of synchronization of LF oscillations in PPGV and HRV (assessed by the S index) with increasing severity of cardiovascular diseases [68,69]. The distributions of the S index in the study groups are shown in Figure 3. The following question arises: what is the reason for the decrease in synchronization of slow oscillations in PPGV and HRV with increasing severity of cardiovascular diseases? We will try to clarify this issue in the following discussion.

It is known that blood microcirculation affects the finger PPG signal, along with the blood filling of the digital arteries [9]. In blood microcirculation, there are LF oscillations that are not associated with autonomic control [70,71,72,73]. We cannot unambiguously determine the contribution of the microcirculatory bed to slow oscillations in PPGV. However, the phenomenon of synchronization of LF oscillations in PPGV and HRV allows for the substantiation of the predominance of cardiovascular autonomic regulation in these oscillations in PPGV. 

Carter et al. reported on the variability and uniqueness of individual human physiological strategies designed to compensate for the progressive decrease in the central blood volume [74]. The combination of these integrated strategies determines the compensatory reserve of cardiovascular regulation. Synchronization of LF oscillations in PPGV and HRV can be considered an indicator of the integration of regulatory mechanisms in the human cardiovascular system. 

In the present study, we showed a progressive decrease in the LF band (in absolute and relative terms) in both HRV and PPGV spectra with increasing severity of cardiovascular diseases (Table 1: healthy subjects → patients with HTN → patients with CAD → patients with PMI → patients with AMI). At the same time, a sharper drop in LF% relative to healthy individuals was observed in PPGV (Figure 1a,b). We revealed solely a minimal and non-significant correlation in the LF band power between the PPGV and HRV spectra (Table 2). Thus, we conclude that LF oscillations in HRV and PPGV are quite independent of each other. 

We can hypothesize that a reduction in the activity of LF oscillations in HRV and PPGV leads to a decrease in their synchronization. The values of all these indices decrease with increasing severity of cardiovascular diseases (Figure 1a,b and Figure 3). However, we detected only a minimal and non-significant direct correlation between the S index and the contribution of LF oscillations to HRV or PPGV (estimated by LF%_HRV_ and LF%_PPGV_), as well as no correlation whatsoever with the power of the LF band (in absolute values) of the HRV spectrum (estimated by LF_HRV_) (Table 2). As a result of multiple analyses, we showed that the synchronization of LF oscillations in PPGV and HRV is associated with only two factors of autonomic control: (1) the contribution of LF oscillations to HRV (estimated by LF%_HRV_) and (2) the ratio of the activity of LF and HF oscillations in PPGV (estimated by LF/HF_PPGV_). However, even these two factors exhibited a minimal and non-significant correlation with the S index (multiple R = 0.34). Hence, the synchronization between LF oscillations in PPGV and HRV is the top independent cardiovascular autonomic parameter, and this finding supports the results we previously obtained in another study on a smaller group of healthy individuals [75]. 

### 4.3. Orthostatic Dynamics of Spectral Indices: PPGV vs. HRV

Two main components with a similar power are characteristic for the LF and HF bands of the HRV spectrum at rest. According to our results and the results of other authors [2], there is a slight predominance of LF oscillations over HF oscillations in the HRV of healthy individuals at rest.

In the orthostatic position, the shift of blood to the lower part of the body determines the decrease in the central BP, thereby affecting the cardiopulmonary and carotid baroreceptors less [76,77]. The arterial baroreflex causes the primary rapid changes in the BP during the passive head-up tilt test [78,79]. We established that in healthy individuals, tilt changes were similar to those recommended for heart rate variability in the guidelines [1]. Increasing the tilt angle also leads to greater sympathetic nerve activity in muscles [80,81,82]. Fu et al. found that this sympathetic activation has a generalized effect on the heart (tachycardia) and blood vessels (vasoconstriction) [83]. As a result, tilt leads to the predominance of LF components (baroreflex and sympathetic activity) and less pronounced HF components (respiratory and parasympathetic activity) in HRV. Contrariwise, the PPGV indicates different spectral changes (increase in HF% and no change in LF%) during the passive head-up tilt test. These differences further confirm the independence of the spectral indices of HRV and PPGV. Thus, tilt results in a constant vascular sympathetic activity against the background of an increase in passive respiratory transmission of the PPG signal in healthy subjects. We did not study the absolute values of the spectral power in the frequency domain of PPGV due to the limitations described above, in the subsection Spectral Analysis of Photoplethysmographic Waveform Variability. However, Giraldo et al. showed smaller BPV indices (including spectral parameters in their absolute values) in the supine position vs. the sitting position [84]. For active standing, Bernardi et al. previously reported an increase in sympathetic activity in both BPV and PPGV [12]. 

Tilt changes of LF synchronization between HRV and PPGV can be considered an indicator of the integrative mechanisms of cardiovascular autonomic regulation. Contradictory tilt changes of LF synchronization between HRV and PPGV, observed by us in healthy individuals and patients with cardiovascular diseases, may indicate a disorder of the integrative function of the central nervous system in the regulation of blood circulation. The most significant differences in synchronization between healthy individuals and patients were found during sympathetic activation in the passive head-up tilt test.

The spectral analysis method for determining BRS is based on the idea that each spontaneous oscillation in BPV leads to an opposite oscillation in HRV due to baroreflex activity [85]. These oscillations in BPV and HRV occur at the same frequencies, about 0.1 Hz for the LF band and in the range of 0.15–0.35 Hz for the HF band [86,87]. 

Baroreflex impairment was often observed in patients with HTN [88,89,90]. To what extent this is a coincidence vs. to what extent it is causal, is a quite speculative issue. It is known that HTN is characterized not only by increased sympathetic activity [89,90], but also by reduced vagal modulation of the sinoatrial node [91] or weaker baroreflex gain [92]. Some studies found that the progressive increase in LF-band power in the HRV spectrum with increasing severity of HTN and the decrease in this power as a result of the passive head-up tilt test are in good agreement with the known deterioration of baroreflex gain in conditions of HTN [93]. There is a chronic shift in the baroreflex threshold towards predominantly higher BP, along with a concomitant decrease in BRS [94]. The cardiovascular system becomes adjusted to the chronic maintenance of higher BP, while BRS is reduced, leading to adverse physiological and prognostic consequences [85].

## 5. Conclusions

The main findings of the study can be summarized as follows:

(1) The PPGV frequency-domain indices (LF%, HF%, and LF/HF) are highly sensitive markers of any cardiovascular disease development, surpassing the HRV indices in this regard;
(2) Changes in all frequency-domain indices and the S index were observed along the following gradient: healthy subjects → patients with HTN → patients with CAD → patients with PMI → patients with AMI;
(3) Similar frequency-domain indices of PPGV and HRV are weakly associated with each other;
(4) The S index is a parameter independent of frequency-domain indices, exhibiting the greatest difference in values between healthy subjects and patients during the passive head-up tilt test.

## 6. Limitations

In our study, we did not designate subgroups by gender since we did not reveal any significant effect of gender on the results obtained during our pilot study analysis. It is known that women have lower values of the standard deviation of normal-to-normal (SDNN) interbeat intervals and LF, but higher HF values compared to men. Gender-based differences weaken with age and decrease during menopause, which may indicate a potential hormonal influence on the autonomic nervous system [95]. Gender-based differences in vascular autonomic control, which may change during the menstrual cycle [96], require further study.

A constant decrease in the time-based parameters and frequency-domain indices of HRV is observed throughout life [97]. It was reported that every 10 years, there is a decrease in the power of the LF and HF spectra of HRV by about 15% [98]. This determines the second important limitation of our study, since the healthy subjects and patients with HTN in our sample were younger than the other three categories of patients. However, it is worth noting that the identified differences between the groups in the HRV and PPGV indices were severalfold rather than within 10–30%, which, in our understanding, partially offset the error associated with age differences. 

We did not analyze the specifics of pharmaceutical treatment in patient groups, but all patients were treated in accordance with the relevant clinical guidelines. Specifics of drug therapy (e.g., early use of beta-blockers in target patients) may have influenced our findings across the different severities of cardiac diseases.

## Figures and Tables

**Table 1:** Cardiovascular autonomic indices (S index, HR, frequency-domain indices of HRV and PPGV) at rest and during the passive head-up tilt test in subjects with various clinical status.

Parameters	Healthy Subjects (n = 53)	Patients			
		HTN (n = 536)	CAD (n = 185)	PMI (n = 104)	AMI (n = 120)
S index_s_, %	37.5 (27.7, 44.9)	23.4 (14.7, 34.7)	21.5 (14.0, 33.3)	18.5 (11.1, 29.4)	16.3 (10.0, 24.6)
S index_u_, %	45.9 (35.4, 57.0) *	20.7 (12.8, 30.6) *	21.3 (11.6, 32.0) *	N/A	N/A
HR_s_, bpm	65 (60, 73)	68 (60, 75)	69 (60, 78)	66 (60, 74)	68 (59, 75)
HR_u_, bpm	86 (77, 97) *	81 (71, 91) *	76 (65, 85) *	N/A	N/A
Spectral indices of HRV					
HF_s_, ms^2^	519 (183, 958)	187 (91, 407)	158 (71, 291)	214 (81, 676)	178 (79, 438)
HF_u_, ms^2^	156 (38, 538) *	108 (48, 190) *	85 (35, 167) *	N/A	N/A
LF_s_, ms^2^	412 (172, 820)	252 (129, 418)	116 (54, 240)	116 (65, 243)	131 (51, 313)
LF_u_, ms^2^	644 (253, 1255)	230 (128, 415)	81 (46, 184) *	N/A	N/A
TP_s_, ms^2^	1238 (713, 2232)	1120 (680, 1877)	807 (414, 1491)	804 (411, 1523)	698 (353, 1559)
TP_u_, ms^2^	967 (532, 2382)	997 (611, 1644) *	524 (326, 1162) *	N/A	N/A
HF%_s_, %	41.0 (26.5, 50.9)	17.8 (10.4, 27.7)	23.0 (13.4, 37.9)	35.8 (16.3, 50.5)	29.6 (17.2, 45.5)
HF%_u_, %	13.5 (8.0, 26.5) *	9.9 (5.9, 16.5) *	14.5 (7.3, 26.9) *	N/A	N/A
LF%_s_, %	32.6 (24.9, 42.5)	21.2 (15.8, 28.2)	17.0 (11.2, 23.1)	16.2 (10.6, 23.1)	18.2 (11.8, 24.1)
LF%_u_, %	51.4 (39.6, 63.9) *	23.7 (16.7, 34.3) *	15.7 (11.4, 22.3)	N/A	N/A
LF/HF_s_, CU	0.83 (0.51, 1.41)	1.28 (0.73, 2.12)	0.76 (0.45, 1.39)	0.50 (0.26, 1.13)	0.64 (0.37, 1.08)
LF/HF_u_, CU	3.72 (1.81, 7.39) *	2.50 (1.27, 4.28) *	1.00 (0.58, 2.60) *	N/A	N/A
Spectral indices of PPGV					
HF%_s_, %	10.3 (4.9, 19.0)	40.4 (12.8, 81.2)	81.4 (39.0, 93.4)	76.1 (40.6, 91.3)	82.5 (38.7, 93.9)
HF%_u_, %	19.8 (8.8, 71.4) *	71.7 (42.7, 88.8) *	79.6 (48.3, 92.5)	N/A	N/A
LF%_s_, %	44.7 (14.4, 58.4)	8.5 (2.3, 23.4)	2.6 (1.6, 6.7)	3.5 (1.4, 8.2)	3.7 (1.4, 9.9)
LF%_u_, %	44.4 (14.7, 60.0)	10.4 (4.2, 25.9)	3.6 (2.3, 7.6)	N/A	N/A
LF/HF_s_, CU	4.79 (1.00, 8.48)	0.28 (0.06, 1.04)	0.04 (0.02, 0.14)	0.06 (0.02, 0.19)	0.04 (0.02, 0.31)
LF/HF_u_, CU	2.00 (0.25, 6.77) *	0.15 (0.06, 0.49) *	0.06 (0.03, 0.17)	N/A	N/A

Note: (1) Data are presented as medians with interquartile ranges, Me (LQ, UQ). (2) * denotes a statistically significant difference (*p* < 0.05) with the value of this parameter in the supine position. (3) Subscripts (_s_) and (_u_) indicate the position of the subject’s body: supine (rest) and upright (passive head-up tilt). (4) HTN, hypertension; CAD, coronary artery disease; MI, myocardial infarction; AMI, acute myocardial infarction; S, relative time (%) of synchronization between low-frequency oscillations in HRV and PPGV; HR, heart rate; bpm, beats per minute; HF, high-frequency (0.15 to 0.40 Hz) spectral band power; LF, low-frequency (0.04 to 0.15 Hz) spectral band power; TP, total spectral power (0 to 0.5 Hz); HF%, ratio (in %) of HF to TP; LF%, ratio (in %) of LF to TP; LF/HF, ratio of LF to HF; CU, conventional unit; N/A, not applicable. (5) Color coding is based on the strength of differences from healthy subjects, calculated based on z-value and *p*-value (post-hoc analysis), as follows: 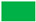: *p* > 0.05; 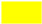: z < 5.0, *p* < 0.05; 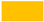: z = 5.0–5.9, *p* < 0.05; 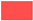: z = 6.0–6.9, *p* < 0.05; 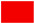: z ≥ 7.0, *p* < 0.05.

**Table 2:** Spearman correlation matrix between the studied cardiovascular autonomic indices.

		HR	HRV						PPGV		
			HF	LF	TP	HF%	LF%	LF/HF	HF%	LF%	LF/HF
S index		0.04	**−0.08**	0.03	−0.03	**−0.11**	**0.13**	**0.17**	**−0.21**	**0.17**	**0.22**
HR			**−0.41**	**−0.36**	**−0.42**	**−0.15**	−0.02	**0.14**	**0.04**	**−0.06**	**−0.07**
HRV	HF			**0.70**	**0.78**	**0.69**	**0.08**	**−0.54**	**0.09**	0.04	0.00
	LF				**0.87**	**0.13**	**0.51**	**0.15**	−0.06	**0.13**	**0.14**
	TP					**0.15**	**0.07**	**−0.08**	0.02	0.06	0.04
	HF%						−0.01	**−0.84**	**0.16**	−0.04	**−0.10**
	LF%							**0.50**	**−0.17**	**0.19**	**0.22**
	LF/HF								**−0.22**	**0.13**	**0.20**
PPGV	HF%									**−0.43**	**−0.84**
	LF%										**0.82**

Note: The data are presented as Spearman’s correlation coefficients (R). Cases of R = [0.68–1.0], R = [0.36–0.67], and R ≤ 0.35 correspond to strong, moderate, and weak (i.e., minimal and non-significant) correlation, respectively. Strong and moderate correlations are highlighted in green and yellow, correspondingly. Coefficients with *p* < 0.05 are marked in bold.

## Data Availability

The parameters presented in this study are available on reasonable request from the corresponding author. The primary data are not publicly available due to the policy of access to clinical data of the Saratov State Medical University.
